# The causal association between obesity and gastric cancer and shared molecular signatures: a large-scale Mendelian randomization and multi-omics analysis

**DOI:** 10.3389/fonc.2023.1091958

**Published:** 2023-10-26

**Authors:** Abao Xing, Henry H. Y. Tong, Songyan Liu, Xiaobing Zhai, Li Yu, Kefeng Li

**Affiliations:** ^1^Centre for Artificial Intelligence Driven Drug Discovery, Faculty of Applied Sciences, Macao Polytechnic University, Macao, Macao SAR, China; ^2^Bioinformatics Department, Guangzhou AoCe Medical Technology Co. Ltd., Guangzhou, China; ^3^Department of Endocrine Rehabilitation, Affiliated Hospital of Liaoning University of Traditional Chinese Medicine, Shenyang, China; ^4^Department of Oncology, Shengjing Hospital of China Medical University, Shenyang, China

**Keywords:** obesity, gastric cancer, causality, shared molecular signatures, multi-omics

## Abstract

**Purpose:**

While observational studies have identified obesity as a potential risk factor for gastric cancer, the causality remains uncertain. This study aimed to evaluate the causal relationship between obesity and gastric cancer and identify the shared molecular signatures linking obesity to gastric cancer.

**Methods:**

A two-sample Mendelian randomization (MR) analysis was conducted using the GWAS data of body fat percentage (exposure, n = 331,117) and gastric cancer (outcome, n = 202,308). Bioinformatics and meta-analysis of multi-omics data were performed to identify key molecules mediating the causality. The meta-analysis of the plasma/serum proteome included 1,662 obese and 3,153 gastric cancer patients. Obesity and gastric cancer-associated genes were identified using seven common gene ontology databases. The transcriptomic data were obtained from TCGA and GEO databases. The Bioinformatic findings were clinically validated in plasma from 220 obese and 400 gastric cancer patients across two hospitals. Finally, structural-based virtual screening (SBVS) was performed to explore the potential FDA-approved drugs targeting the identified mediating molecules.

**Results:**

The MR analysis revealed a significant causal association between obesity and gastric cancer (IVW, OR = 1.37, 95% CI:1.12-1.69, *P* = 0.0028), without pleiotropy or heterogeneity. Bioinformatic and meta-analysis of multi-omics data revealed shared TNF, PI3K-AKT, and cytokine signaling dysregulation, with significant upregulation of AKT1, IL-6, and TNF. The clinical study confirmed widespread upregulation of systemic inflammatory markers in the plasma of both diseases. SBVS identified six novel potent AKT1 inhibitors, including the dietary supplement adenosine, representing a potentially preventive drug with low toxicity.

**Conclusion:**

Obesity causally increases gastric cancer, likely mediated by persistent AKT1/IL-6/TNF upregulation. As a potential AKT1 inhibitor, adenosine may mitigate the obesity-to-gastric cancer transition. These findings could inform preventive drug development to reduce gastric cancer risk in obesity.

## 1 Introduction

With the rapid development of the social economy, improved living standards, and changes in living habits, the number of obese people is increasing globally. According to a recent World Health Organization (WHO) report, 39% of adults aged 18 years and over were overweight in 2016, and 13% were obese worldwide (1). Obesity is recognized as a chronic, progressive disease requiring long-term management (2). Compared to those with normal weight, individuals who are overweight and obese are more likely to develop a number of potentially serious health conditions, including type 2 diabetes mellitus, nonalcoholic fatty liver disease, hypertension, myocardial infarction, stroke, dementia, osteoarthritis and obstructive sleep apnea (3–6).

In recent years, cancers associated with overweight and obesity have also been noticed. It has been reported that obesity might increase the incidence of several types of cancer (7–10), including gastric, colorectal, bladder, liver, kidney, pancreatic, and breast cancers. Furthermore, obesity and overweight are associated with increased risks of cancer mortality (11). Unlike other cancer types, for gastric cancer specifically, the relationship remains controversial and not fully understood. While some cohort studies have indicated a positive association between high body mass index (BMI) and increased gastric cancer risk (12–14), others found no statistically significant relationship (15). In the past few years, systematic reviews addressing the BMI-gastric cancer link have yielded inconsistent results (16, 17). A meta-analysis published in 2023 suggested a positive association between excess body weight and the risk of gastric cancer (18). However, it remains uncertain whether the observed association reflects a direct causal effect of BMI on gastric carcinogenesis, or stems from confounding or biases inherent in conventional epidemiological studies. For instance, the observational BMI-cancer association may be biased by smoking, and diet, which can independently influence both BMI and cancer risk (8).

Mendelian randomization (MR) utilizes genetic variants as instrumental variables to make causal inferences between exposures and outcomes. Since genotype is presumed to be randomly allocated at conception, confounding factors are anticipated to distribute equally among different genotypes. Therefore, compared to traditional observations and randomized controlled trials, MR reduces the issues of potential confounding, reverse causation, and feasibility (19). To date, MR has been employed to investigate the potential causal relationships between obesity and a variety of diseases, including obesity (20), cardiovascular diseases (21), depression (22) and thyroid cancer (23). A prior small-scale MR study with limited BMI-related single nucleotide polymorphisms (SNPs) (<50) suggested putative gastric cancer risk from obesity (24). However, large-scale MR incorporating more exposure and outcome genetic variants is warranted to reliably determine causality.

Furthermore, the underlying mechanisms that causally increase the risk of gastric cancer in obese patients and the potential pharmaceutical interventions have not been fully explored. Existing research indicates that several obesity-related risk factors and certain signaling pathways are hypothesized to play important roles in the inception and progression of cancers (25). Nonetheless, the biological mechanisms and the relationships involving obesity and gastric cancer are intricate and remain largely unclear. This complexity is reflected in an array of influencing factors, including obesity-associated hormones and adipokines, growth factors, energy balance regulation, inflammatory processes, and multiple signaling pathways that affect cancer progression (26, 27). Possible mechanisms linking obesity with gastric cancer encompass obesity-associated insulin resistance, abnormally elevated blood levels of insulin-like growth factor (IGF), and associated levels of adipokines such as adiponectin (APN), leptin, steroid hormones and cytokines. Each of these elements alters the nutritional milieu, potentially fostering an environment conducive to tumor initiation and progression (28).

To address the aforementioned gaps, in this study, we conducted a large-scale, two-sample MR analysis to explore the causal association between obesity and gastric cancer using the genome-wide association analysis (GWAS) data from 202,308 East Asian individuals and 8,885,324 SNPs (Gastric cancer). Furthermore, integrated bioinformatics and meta-analyses of multi-omics data were performed to identify the key molecular signatures connecting obesity to elevated gastric cancer risk. A retrospective multi-center cohort study was then conducted to validate the in silico meta-analysis and bioinformatics. Finally, structure-based virtual docking analysis was performed to screen the potential FDA-approved drugs that target the mediating molecules, which may mitigate the obesity-to-gastric cancer transition therapeutically. This multifaceted study elucidates putative causal mechanisms underlying obesity-associated gastric cancer and nominates targeted therapeutic strategies to reduce gastric cancer incidence in obese populations.

## 2 Materials and methods

### 2.1 Mendelian randomization analysis

The GWAS summary statistic data for body fat percentage (n = 331,117) and gastric cancer (n = 202,308) were obtained from previously published studies (29) and used as exposure and outcome datasets, respectively. All included GWAS had F-statistics > 10, satisfying MR assumptions.

The SNPs associated with the exposures at genome-wide significance (*P* < 5 × 10^−8^) were selected as instrumental variables (IVs). To ensure the independence, IVs were pruned for linkage disequilibrium using LD clumping (r^2^ < 0.001, distance 10,000 kb). To investigate the causality between obesity and gastric cancer, the random-effect inverse variance weighted (IVW) method was utilized as the primary analysis in the MR analyses. We performed the Cochran Q test to assess the heterogeneity. To evaluate the robustness of the MR estimates, we also compared the IVW estimation with other MR models, including maximum likelihood, and MR-Egger regression. We also utilized the intercept term derived from MR-Egger to assess the horizontal pleiotropy. All MR analyses were conducted with the “TwoSampleMR” and “Mendelian Randomization” packages in the R software (version 4.3.1).

### 2.2 Meta-analysis of the plasma/serum proteome

Search strategy: The present meta-analysis followed the guidelines of the PRISMA 2020 Statement. We performed a comprehensive literature search of articles through PubMed without date limitation. Searches were updated to May 17, 2023 without language restrictions. The main search terms for obesity were as follows: Obesity AND (plasma OR blood OR serum) AND (proteomics OR proteome). The search terms for gastric cancer were “stomach neoplasms”[MeSH Terms] OR “Neoplasm, Stomach” OR “Stomach Neoplasm” OR “Neoplasms, Stomach” OR “Gastric Neoplasms” OR “Gastric Neoplasm” OR “Neoplasm, Gastric” OR “Neoplasms, Gastric” OR “Cancer of Stomach” OR “Stomach Cancers” OR “Gastric Cancer” OR “Cancer, Gastric” OR “Cancers, Gastric” OR “Gastric Cancers” OR “Stomach Cancer” OR “Cancer, Stomach” OR “Cancers, Stomach” OR “Cancer of the Stomach” OR “Gastric Cancer, Familial Diffuse”) AND (plasma OR blood OR serum) AND (proteomics OR proteome).

Inclusion and exclusion criteria: The inclusion criteria for selecting the studies for this meta-analysis were as follows (1): proteomic analysis of obese/gastric cancer patients (2), plasma/serum samples (3), case-control comparison, and (4) All study designs. The exclusion criteria were (1): animal studies (2), unidentified proteins, and (3) meta-analyses, meeting abstracts, letters to the editor, case reports, and reviews. The flow charts of the meta-analysis selection process are listed in **Figures S1**, **S2**.

Data extraction and synthesis: Two authors (AX and H.H.Y.T) independently evaluated all possible articles and extracted relevant information. Any disagreements were resolved by a third author (X.Z). The extracted data from each study included sample sizes, significantly altered protein names/UniProt IDs, fold changes (FC, case/control), adjusted *P* values, and the area under the receiver operating characteristic (ROC) curve (AUROCs). This information can be found in **Tables S1**, **S2**. We performed cross-data quality checks between reviewers at each step and reviewed all the references included after constructing the dataset.

Meta-analysis: To perform the meta-analysis, we standardized the effect size for each protein to fold change (Case/control) and only included proteins with FDR adjusted *P* values < 0.05 for the analysis. Gene IDs (Entrez IDs) were converted from protein Uniport IDs using HGNC (https://www.genenames.org/). Pathway enrichment analysis was conducted using DataVis Builder (http://bioinformatics.vip), and the interactome of the enriched pathways was visualized through Cytoscape 3.10.0. Higgins I-squared (I^2^) statistic was used to assess the heterogeneity across the included studies. A random-effect meta-analysis was used for I^2^>50%, otherwise a fixed-effect model was performed. Meta-analysis was performed in Stata 17.0, and forest plots were created.

### 2.3 Identification of obesity and gastric cancer associated genes

First, the MeSH IDs for obesity (MeSH ID: D009765) and gastric cancer (MeSH ID: D013274) were obtained from NCBI. Next, Corresponding keywords were identified using the MeSH database: Obesity: D009765, Adiposity, Adiposis; Gastric cancer: D013274, Stomach Neoplasm, Stomach Carcinoma, Stomach Cancer, Gastric Neoplasm, Gastric Cancer, Gastric Carcinoma. Disease-associated genes were retrieved from CTD, TTD, OMIM, GeneCards, MalaCards, DisGeNET and DrugBank using the keywords, from each database’s release to May 25, 2022.

The retrieved disease-associated genes were then filtered as follows: CTD: InferenceScore ≥ 50, GeneCards and MalaCards: Score ≥ 10 in GeneCards and MalaCards databases, and DisGeNET: Score ≥ 0.3. After conversion to gene IDs using UniProt and Entrez, duplicated and invalid entries were removed. Genes with ≥4 occurrences were retained.

### 2.4 Construction of obesity and gastric cancer gene interaction networks

Disease-associated genes identified in the previous step were used as seed genes to construct interaction networks. Protein-protein and gene-regulatory interactions were obtained from HPRD, BioGRID, and KEGG. For HPRD, direct interactors of seed genes were selected to build a background disease network. In BioGRID, interactions with ≥2 counts were retained as the background network. From KEGG, protein-protein (PPrel) and gene expression (Gerel) interactions were integrated. The Jaccard indexwas used to assess the similarity and diversity of two gene interaction networks. It is calculated as the intersection size divided by the union size (J = |A∩B|/|A∪B|).

Cytoscape v3.9.1 and the jActiveModules plugin were used to mine bioactive modules from the integrated obesity-gastric cancer network (NHOGC). Differentially expressed genes (DEGs) from obesity (GSE9624) and gastric cancer (GSE54129, GSE29998) GEO datasets were integrated into NHOGC. The top 5 bioactive modules per dataset were merged into independent active networks for each disease. The networks were then analyzed using Cytoscape MCODE plugin (Degree Cutoff = 2, Node Score Cutoff = 0.2, K-Core = 2, Max. Depth = 100) to identify molecular complexes.

### 2.5 Analysis of AKT1, IL-6, TNF gene expression, and associated pathways in obesity and gastric cancer

GEO database: Microarray gene expression data were collected from GEO for obesity and gastric cancer. Inclusion criteria were (1): human case-control studies (2); gene expression profiling (3), ≥3 case and control samples per study (4), detailed methods and probe annotation files. After screening, the following datasets were selected: one dataset for obesity (GSE9624) and two datasets for gastric cancer (GSE54129, and GSE29998). Further details can be found in **Supplementary Material/Method**.

TCGA database: TCGA clinical data was used to categorize samples into the obese (BMI ≥ 30, n = 113) and the normal weight (18.5 < BMI ≤ 24.9, n = 96) groups. For gastric cancer, Level 3 RNA-seq data was downloaded from UCSC XENA (https://xenabrowser.net/datapages/). A total of 417 samples were obtained, including 380 gastric cancer and 37 adjacent normal tissues. Patients without BMI values were excluded (see details in **Supplementary Material/Method**).

### 2.6 Multi-center, retrospective clinical cohort validation

To validate the results of our meta-analysis and bioinformatic analysis, we conducted a retrospective cohort study using data from two hospitals: Shengjing Hospital of China Medical University (SJH) and Affiliated Hospital of Liaoning University of Traditional Chinese Medicine (LUTCMH). The study protocols were approved by the respective Institutional Review Boards (IRBs) (SJH IRB # sj-2023-c411 and LUTCMH IRB # LUTCM-Endo-20230122).

Inclusion criteria were untreated obesity/gastric cancer patients aged 18-85 years at these centers between June 2020-June 2023. Exclusion criteria were co-existing diseases, prior treatment, or pregnancy. A total of 220 obesity and 400 gastric cancer patients were enrolled (80 obesity, and 290 gastric cancer from SJH). Medical records were reviewed to extract baseline complete blood count (CBC), and analyzed the following systematic inflammatory markers: white blood cell count (WBC), percentage of neutrophils (%NEUT), percentage of eosinophils (%EOS), percentage of basophils (%BASO); percentage of monocytes (%MONO), percentage of lymphocytes (%LYMPH), neutrophil count (#NEUT), eosinophil count (#EOS), basophil count (#BASO), monocyte count (#MONO), lymphocyte count (#LYMPH), red blood cell count (RBC), hemoglobin (HGB), hematocrit (HCT), mean corpuscular volume (MCV), mean corpuscular hemoglobin (MCH), mean corpuscular hemoglobin concentration (MCHC), red cell distribution width coefficient of variation (RDW-CV), red cell distribution width standard deviation (RDW-SD), platelet count (PLT), platelet crit (PCT), mean platelet volume (MPV), platelet distribution width (PDW), platelet large cell ratio (P-LCR). All participants provided written informed consent prior to blood collection.

### 2.7 Structure-based virtual screening

To identify the compounds with the favorable interaction with the AKT1, the 3D crystal structure of AKT1(PDB ID: 3MV5) was used for docking-based virtual screening. Through molecular docking simulation of the binding pocket of AKT1, 1729 FDA approved drugs were screened. Protein preparation: The AKT1 structure was prepared using the Schrödinger Protein Preparation Wizard (Schrödinger, LLC, New York, NY) by removing water molecules, optimizing bonds, and adding hydrogens. Ligand preparation: All FDA-approved ligands were prepared using the LigPrep module in the Schrödinger package to generate the stereoisomers of each ligand. Receptor grid generation: The AKT1 binding site grid was defined using Schrödinger’s Receptor Grid Generation. Molecular docking: Prepared ligands were docked using Schrödinger’s SP docking protocol. Docking poses were ranked by docking score. All docking results were sorted from the lowest to highest of the docking score. Validation: The co-crystallized inhibitor was re-docked into the AKT1 site to evaluate binding reproducibility. Visualization of docking results was performed by PyMOL.

### 2.8 Statistical analysis

Adult BMI classifications were: underweight (<18.5 kg/m^2^), normal weight (18.5 to 24.9 kg/m^2^), overweight (25 to 29.9 kg/m^2^), and obese (≥30 kg/m^2^). Data are presented as mean ± standard deviation (SD) or median and interquartile range (IRQ), depending on the distribution. Differences between obesity and gastric cancer groups were analyzed using either Student’s t-test or the Mann-Whitney U test. The proportions between the two groups were compared using the two-proportion z-test. All statistical analyses, unless otherwise specified, were conducted using R version 4.3.1. A *P* < 0.05 was considered statistically significant.

## 3 Results

### 3.1 Overview of the study design

**Figure 1:**
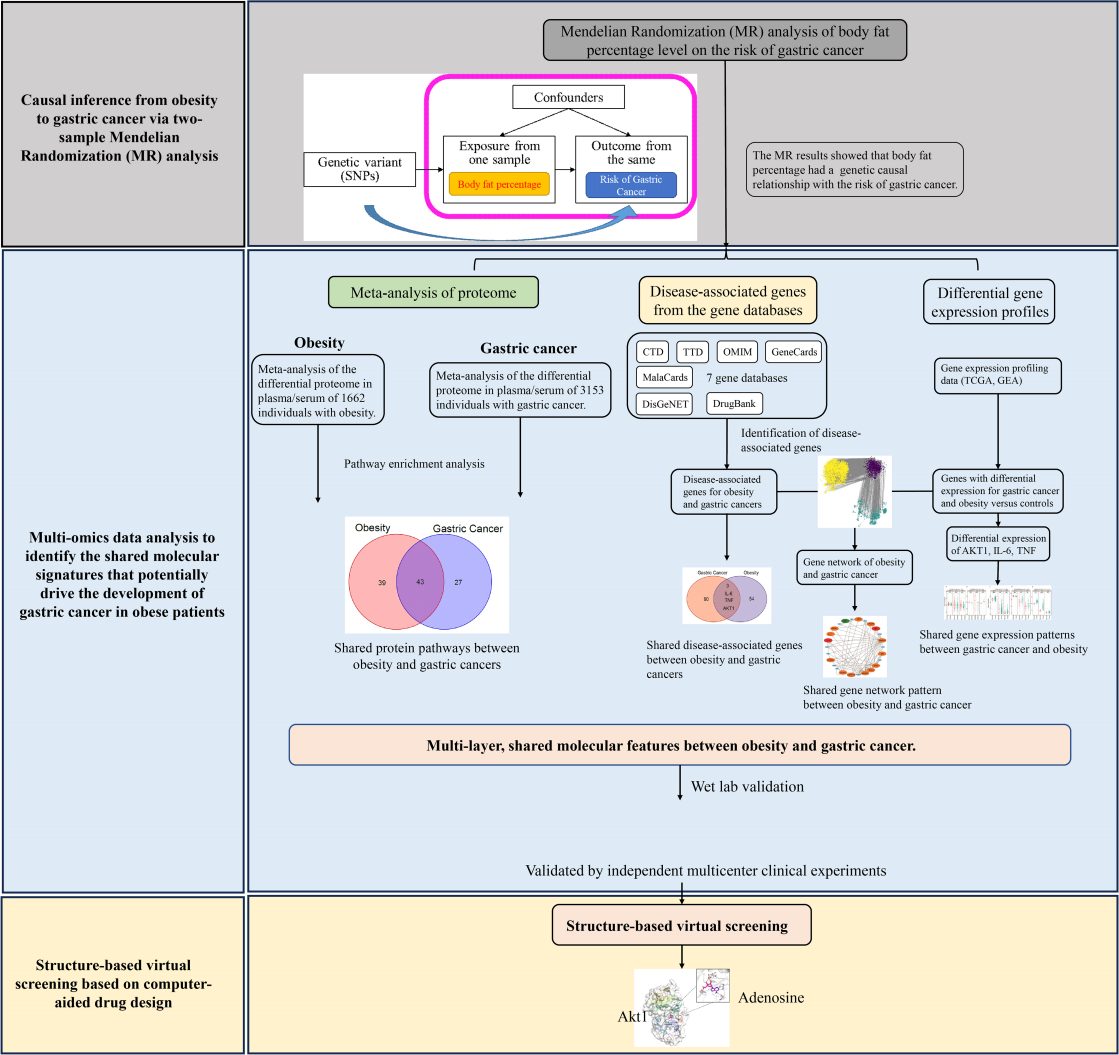
The overall design of this study.

The overview of the study design for this work is shown in **Figure 1**. Briefly, we first conducted Mendelian randomization analysis to explore the causal relationship between obesity and gastric cancer. To identify the molecular mechanisms linking obesity to gastric cancer, we performed the bioinformatic analyses and meta-analyses of multi-omics data. For the proteome, we meta-analyzed all published proteomics studies to characterize and compare the alternations in the plasma/serum proteome between patients with obesity and gastric cancer. At the genomic level, we leveraged seven major gene ontology databases to pinpoint obesity- and cancer-associated genes. Transcriptomic data from TCGA and GEO databases were analyzed to delineate shared dysregulated gene expression profiles in gastric cancer and obesity. Furthermore, the bioinformatics and meta-analysis findings were validated using multi-center, retrospective clinical cohorts. Finally, structural-based virtual screening (SBVS) was implemented to explore prospective FDA-approved drugs targeting the identified putative mediating molecules, which may prevent the transition from obesity to gastric cancer.

### 3.2 MR analysis of body fat percentage on gastric cancer risk

**Figure 2:**
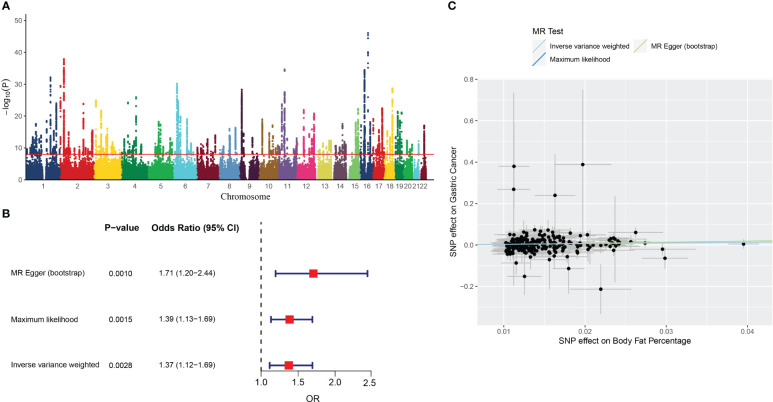
The results of MR analysis. **(A)** Manhattan plot showing distribution of p-values from genome-wide association study of body fat percentage (ukb-a-264). **(B)** Forest plot of MR analysis of the body fat percentage and gastric cancer. **(C)** Individual estimates about the causal effect of Body fat percentage on Gastric Cancer. The X-axis shows the single nucleotide polymorphism (SNP) effect and standard error (SE) on each of the 261 body fat percentage SNPs. The Y-axis shows the SNP effect and SE on gastric cancer. Analyses were conducted by using the conventional IVW, MR-Egger (bootstrap), Maximum likelihood. The slope of each line corresponding to the estimated MR effect per method.

In total, 24,630 genetic variants associated with body fat percentage reached genome-wide significance (*P <*5×10^−8^) (**Figure 2A**). of these, 261 SNPs were selected as the IVs (**Table S3**). The F-statistics for the IVs ranged from 17.15036 to 248.93198, all exceeding 10, indicating the IVs were not biased by weak instruments (**Table S4**).

The IVW analysis showed that the genetic changes in the body fat percentage were statistically associated with the risk of gastric cancer (OR =1.37, 95% CI: 1.12-1.69, *P* = 0.0028, **Figure 2B**), with no evidence of heterogeneity among IVs (Q = 239.7314, *P* = 0.1385).

The causality between body fat percentage and gastric cancer was also confirmed by other MR models, including the MR-Egger (bootstrap) (OR = 1.71, 95% CI: 1.19-2.45, *P* = 0.004), and maximum likelihood [OR = 1.39, 95% CI: 1.13-1.69, *P* = 0.0015) (**Figure 2B**). The scatter plot and trend line showed the consistent trend of causal relationship between obesity and gastric cancer for all three MR models (**Figure 2C**). The MR Egger test showed no indication of horizontal pleiotropy (*P* = 0.39).

### 3.3 The shared genes associated with obesity and gastric cancer

**Figure 3:**
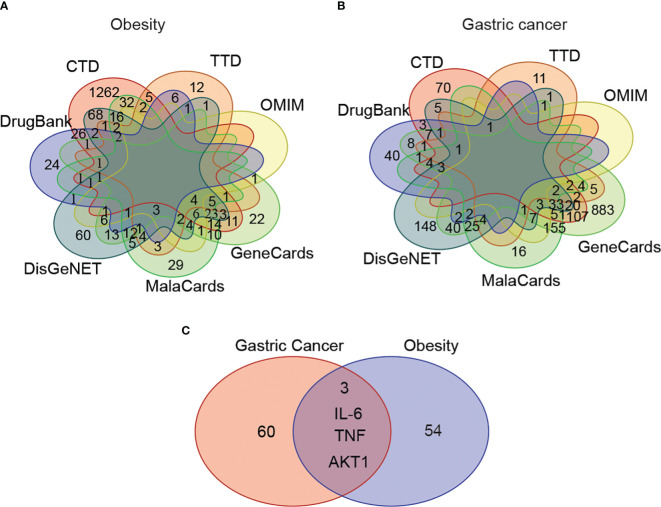
The shared disease-associated genes between obesity and gastric cancer across seven common gene annotation databases. **(A)** The obesity-associated genes across seven gene annotation databases. **(B)** The gastric cancer-associated genes among seven common gene databases. **(C)** The shared disease-associated genes between obesity and gastric cancer.

The disease-associated genes related to obesity and gastric cancer were explored across seven major gene ontology databases, including CTD, TTD, OMIM, GeneCards, MalaCards, DisGeNET, and DrugBank. After sorting, ID conversion, and removing the duplicates and invalid values, we identified 1,712 obesity-related genes and 1,669 gastric-cancer-related genes. The overlap of these genes between databases for each disease is shown in **Figures 3A, B**.

We observed variability in the content and number of relevant genes curated across databases. To address this issue, we focused on high-confidence genes appearing in ≥4 databases concurrently for subsequent analyses. This yielded 57 target genes associated with obesity and 63 with gastric cancer. Among these, 3 genes - AKT1, IL-6, and TNF - were shared between the obesity and gastric cancer sets (**Figure 3C**).

### 3.4 The shared gene interaction networks in obesity and gastric cancer

Using the disease-associated genes from section 3.3, we constructed obesity and gastric cancer gene interaction networks using three major gene interactome databases: HPRD, BioGRID, and KEGG. The obesity network comprised 732, 976, and 1,044 edges in HPRD, BioGRID, and KEGG, respectively (**Figure S5A**). The gastric cancer network contained 2,151, 4,681, and 2,386 edges, respectively (**Figure S5B**). Integrating both networks yielded a combined human obesity-gastric cancer network (NHOGC) (**Figure S5C**). Interestingly, strong associations existed between the diseases at interaction network level, with the Jaccard similarity coefficients of 23.5% for nodes and 6.1% for edges.

**Figure 4:**
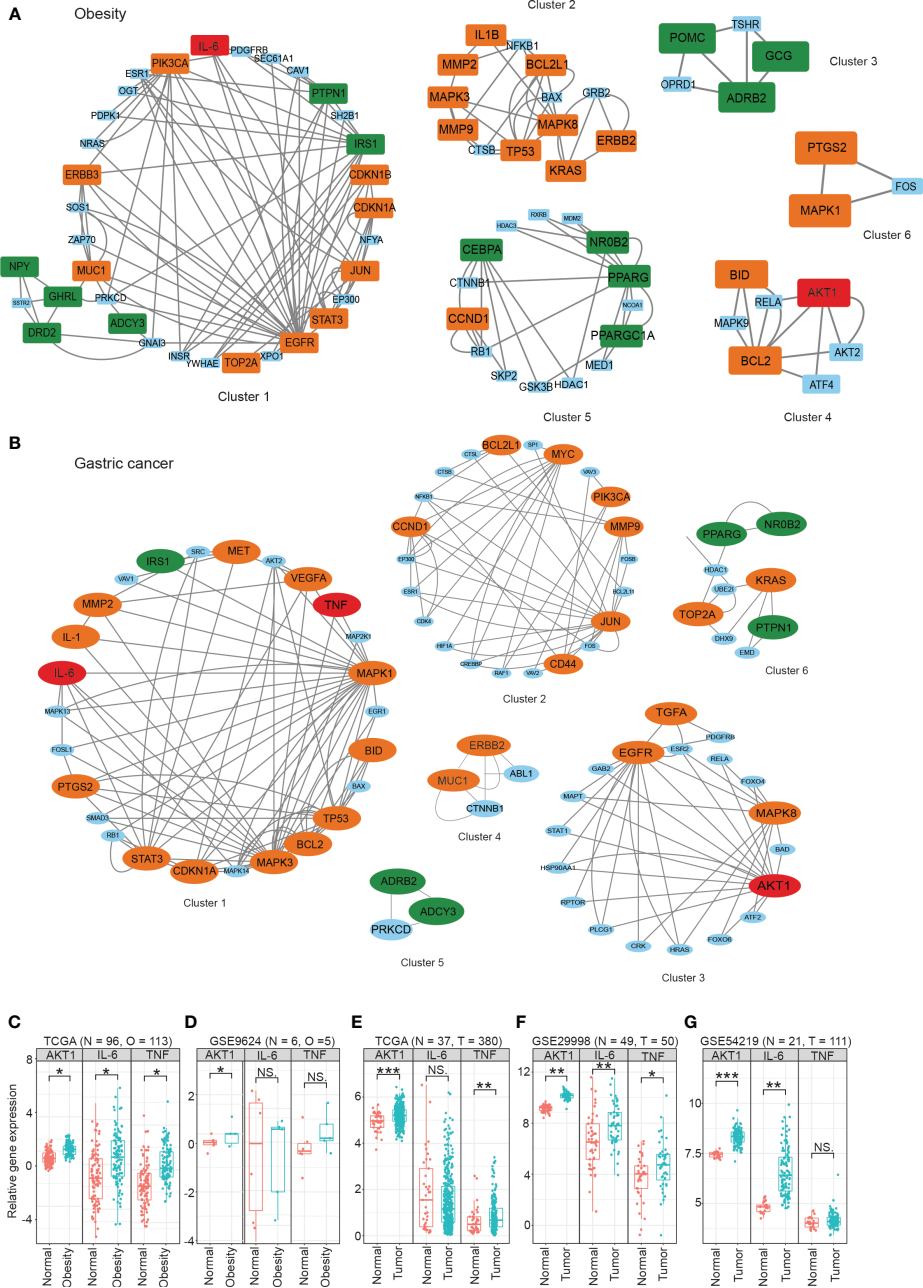
The highly interconnected modules in active gene interaction networks of obesity **(A)** and gastric cancer **(B)**. The data were obtained from publicly available databases, including TCGA **(C, E)**, GEO **(D, F, G)**, For obesity, the overall fold changes (FCs) of the protein expression levels in three signaling pathways were meta-analyzed using the proteomics data from plasma/serum **(A)**, while gene expression analysis was performed using omental adipose tissue **(C, D)**. For gastric cancer, the overall fold changes of protein expression in three signaling pathways were meta-analyzed using the proteomics data from plasma/serum **(B)**, and gene expression analysis was performed using tumor tissue samples **(E–G)**. Statistical significance was determined using the student’s t-test or Mann-Whitney U test, with *P < 0.05, **P < 0.01, and ***P < 0.001 denoting significance levels. The MCODE plugin in Cytoscape was used to identify the highly interconnected regions (clusters) in the constructed bioactive gene interaction networks of obesity and gastric cancer. These clusters often play critical roles in biological systems. NS means not significant.

To better understand the modularity in biological networks and explore their biological functions, we integrated gene expression data from transcriptomic analysis with NHOGC to create bioactive subnetworks for obesity and gastric cancer. The resulting active disease networks contained 1,338 nodes and 2,685 edges for obesity (**Figure S6A**) and 3,045 nodes and 5,947 edges for gastric cancer (**Figure S6B**), respectively. We further used the MCODE plugin in Cytoscape to identify the highly interconnected regions (clusters) in the constructed bioactive gene interaction networks of obesity and gastric cancer. These clusters often play critical roles in biological systems. We identified six highly interconnected gene clusters for obesity (**Figure 4A**) and six gastric cancer-related gene clusters (**Figure 4B**). Strikingly, 36.8% of obesity and 39.7% of cancer genes from Section 3.2 fell within these highly connected clusters, with substantial overlap between diseases. For instance, the obesity cluster 1 contained ten cancer-linked genes (**Figure 4A**). Reciprocally, cancer clusters included obesity genes (**Figure 4B**). Crucially, AKT1, IL-6, and TNF were present in the active gene interaction clusters of both diseases.

### 3.5 Analysis of AKT1, IL-6, and TNF gene expression in obesity and gastric cancer transcriptomics

At the transcriptomic level, we observed significantly increased expression of AKT1 in both the omental adipose tissue from obese patients and the tumor tissue of gastric cancer patients compared to their respective controls (**Figures 4C–G**). However, some heterogeneity was noted for IL-6 and TNF expression across cohorts, possibly due to differences in subject enrollment criteria and experimental conditions.

### 3.6 Meta-analysis of plasma/serum proteomics reveals shared alterations in obesity and gastric cancer

We performed a systematic meta-analysis of plasma/serum proteomics studies in obesity and gastric cancer. For obesity, 13 studies were included (n=1662, **Figure S1**). For gastric cancer, 28 studies were analyzed (n=3153, **Figure S2**). **Tables S1**, **S2** provides the essential characteristics of the studies included in our analysis. In obesity, 161 differentially expressed proteins were identified, mapping to 21 significantly enriched pathways (≥3 proteins/pathway) (**Figure S3A**). In gastric cancer, 158 proteins across 14 pathways were altered (**Figure S3A** and **Table S2**). Of these, 43 proteins were commonly differentially expressed in both obesity and gastric cancer (**Figure S3B**). Notably, 43 dysregulated proteins overlapped between diseases, largely mapping to inflammation and immune pathways like TNF, PI3K-AKT, and cytokine signaling (**Figures S3C**, **S4**).

**Figure 5:**
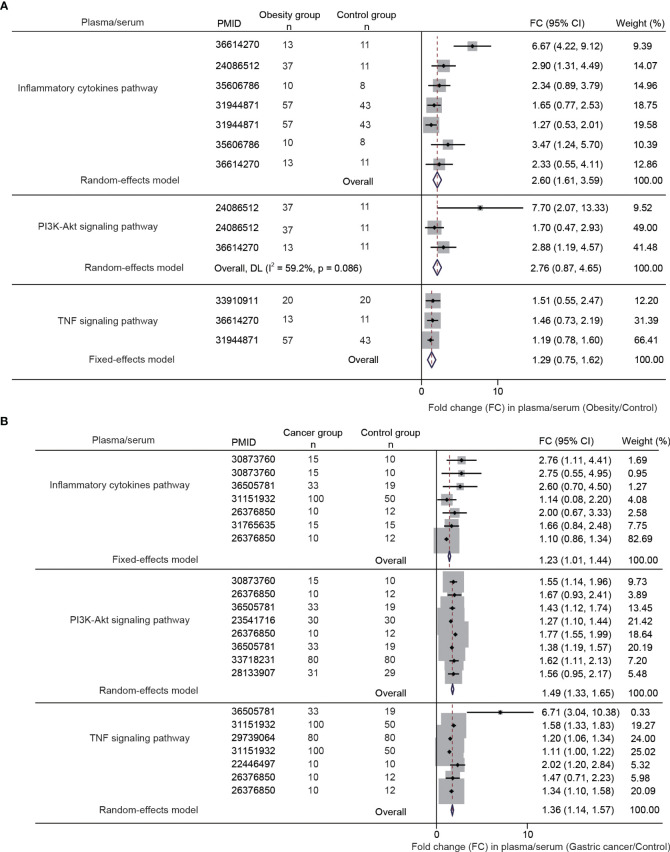
The differential gene and protein expression of PI3K-AKT signaling pathway, inflammatory cytokines pathway, and TNF signaling pathway between obesity and gastric cancer compared with the normal controls. The data were obtained from a meta-analysis of the published studies [**(A, B**), the inclusion flowchart is listed in **Figures S1** and **S2**].

We examined the combined fold changes (FC) of the shared pathways in obesity and cancer versus controls. In obesity, PI3K-AKT (FC 2.76, 95% CI: 0.87-4.65), inflammatory cytokines (FC 2.60, 95% CI: 1.61-3.59), and TNF signaling (FC 1.29, 95% CI: 0.75-1.62) were upregulated (**Figure 5A**). Similarly, these pathways were overexpressed in gastric cancer - PI3K-AKT (FC 1.23, 95% CI: 1.01-1.44), inflammatory cytokines (FC: 1.49, 95% CI: 1.33-1.65), TNF signaling (FC 1.36, 95% CI: 1.14-1.57) (**Figure 5B**).

### 3.7 Multi-center clinical cohort validation

**Figure 6:**
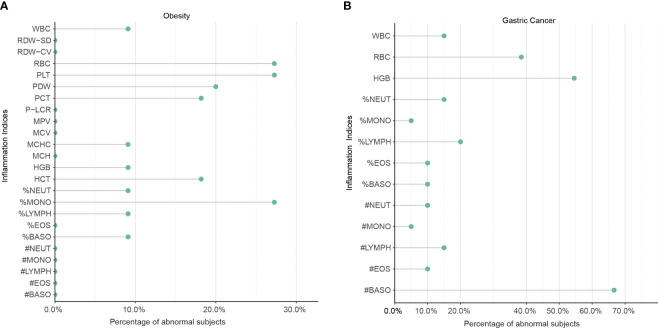
The shared upregulation of systematic inflammatory markers between obesity and gastric cancers was validated by a multi-center clinical study. **(A)** Abnormal inflammation parameters in patients with obesity (n = 220). **(B)** Abnormal inflammation parameters in patients with gastric cancer (n = 400). A total of 220 patients with obesity and 400 patients with gastric cancer were enrolled in two centers. The number of patients with abnormal systemic inflammatory markers in the complete blood cell count (CBC) was analyzed. WBC, White Blood Cell Count; %NEUT, Percentage of Neutrophils; %EOS, Percentage of Eosinophils; %BASO, Percentage of Basophils; %MONO, Percentage of Monocytes; %LYMPH, Percentage of Lymphocytes; #NEUT, Neutrophil Count; #EOS, Eosinophil Count; #BASO, Basophil Count; #MONO, Monocyte Count; #LYMPH, Lymphocyte Count; RBC, Red Blood Cell Count; HGB, Hemoglobin; HCT, Hematocrit; MCV, Mean Corpuscular Volume; MCH, Mean Corpuscular Hemoglobin; MCHC, Mean Corpuscular Hemoglobin Concentration; RDW-CV, Red Cell Distribution Width Coefficient of Variation; RDW-SD, Red Cell Distribution Width Standard Deviation; PLT, Platelet Count; PCT, Platelet Crit; MPV, Mean Platelet Volume; PDW, Platelet Distribution Width; P-LCR, Platelet Large Cell Ratio.

To validate the meta-analysis and bioinformatics findings, a retrospective multi-center cohort study was conducted. 220 obesity and 400 gastric cancer patients were enrolled across two hospitals (**Table 1**). We examined complete blood count markers to assess shared chronic inflammation and immune activation in both diseases. Notably, widespread abnormalities were observed in inflammatory markers including monocyte percentage, white blood cell count, and basophil count in whole blood of obesity and gastric cancer groups (**Figures 6A, B**). Overall, the clinical data corroborated the presence of shared chronic inflammation and immune activation in obesity and gastric cancer evident across multi-omics layers - from genes and networks to expression and plasma proteins. The collective multi-level evidence points to interconnected inflammatory and immune dysregulation in the pathophysiological links between obesity and gastric cancer risk.

**Table 1:** Patient characteristics in the independent clinical validation study.

Patient characteristics	Obesity (n = 220)	Gastric cancer (n = 400)	*P* value
Gender, male (%)	160 (72.7%)	300 (75%)	1.00
Age (median [IQR])	48.0 [40.0, 54.5]	65.0 [57.0, 69.3]	0.042
BMI (mean ± SD)	32.9 ± 7.5	23.4 ± 5.8	0.003
WBC (median [IQR])	7.56 [6.48, 7.79]	6.52 [5.45, 8.05]	0.302
%NEUT (median [IQR])	57.7 [55.4, 61.7]	64.4 [55.1, 70.9]	0.154
%EOS (median [IQR])	2.1 [1.2, 2.4]	1.5 [1.0, 2.2]	0.374
%BASO (mean ± SD)	0.62 ± 0.40	0.60 ± 0.29	0.917
%MONO (median [IQR])	6.9 [4.9, 7.9]	7.5 [6.2, 9.2]	0.223
%LYMPH (median [IQR])	32.5 [27.4, 36.3]	23.3 [19.9, 32.4]	0.047
#NEUT (median [IQR])	4.65 [3.38, 5.08]	4.50 [2.70, 5.35]	0.836
#EOS (mean ± SD)	0.15 ± 0.07	0.12 ± 0.09	0.414
#BASO (mean ± SD)	0.04 ± 0.02	0.02 ± 0.04	0.110
#MONO (median [IQR])	0.52 [0.40, 0.58]	0.50 [0.40, 0.60]	0.771
#LYMPH (median [IQR])	2.42 [1.95, 2.64]	1.50 [1.17, 2.02]	0.004
RBC (mean ± SD)	4.93 ± 0.62	4.30 ± 0.92	0.067
HGB (mean ± SD)	148.3 ± 19.7	109.7 ± 41.9	0.012
HCT (median [IQR])	45.0 [40.3, 46.7]	38.6 [34.8, 42.6]	0.063
MCV (median [IQR])	88.9 [86.8, 90.5]	86.3 [81.8, 89.2]	0.295
MCH (median [IQR])	30.0 [28.6, 31.2]	28.8 [26.9, 30.7]	0.601
MCHC (mean ± SD)	339.9 ± 13.7	284.3 ± 42.4	0.001
RDW-CV (mean ± SD)	12.9 ± 0.87	16.5 ± 3.84	0.009
RDW-SD (mean ± SD)	41.4 ± 2.5	49.8 ± 7.80	0.006
PLT (median [IQR])	220.0 [207.5, 301.5]	312.5 [253.0, 379.5]	0.433
PCT (median [IQR])	0.22 [0.21, 0.27]	0.26 [0.18, 0.23]	0.512
MPV (median [IQR])	9.85 [8.72, 10.5]	9.73 [8.58, 10.7]	0.347
PDW (mean ± SD)	13.5 ± 2.94	9.73 ± 0.25	0.057
P-LCR (median [IQR])	23.9 [15.6, 29.2]	25.1 [14.7, 27.6]	0.714

Data are mean ± standard deviation (SD) or median and interquartile range (IRQ) depending on the distribution. Differences between obesity and gastric cancer groups were analyzed using either Student’s t-tests or Mann–Whitney U tests. The proportions between the two groups were analyzed using the two-proportion z-test. P < 0.05 was statistically significant.

BMI, Body Mass Index; WBC, White Blood Cell Count; %NEUT, Percentage of Neutrophils; %EOS, Percentage of Eosinophils; %BASO, Percentage of Basophils; %MONO, Percentage of Monocytes; %LYMPH, Percentage of Lymphocytes; #NEUT, Neutrophil Count; #EOS, Eosinophil Count; #BASO, Basophil Count; #MONO, Monocyte Count; #LYMPH, Lymphocyte Count; RBC, Red Blood Cell Count; HGB, Hemoglobin; HCT, Hematocrit; MCV, Mean Corpuscular Volume; MCH, Mean Corpuscular Hemoglobin; MCHC, Mean Corpuscular Hemoglobin Concentration; RDW-CV, Red Cell Distribution Width Coefficient of Variation; RDW-SD, Red Cell Distribution Width Standard Deviation; PLT, Platelet Count; PCT, Platelet Crit; MPV, Mean Platelet Volume; PDW, Platelet Distribution Width; P-LCR, Platelet Large Cell Ratio.

### 3.8 Results of structural-based virtual docking

**Figure 7:**
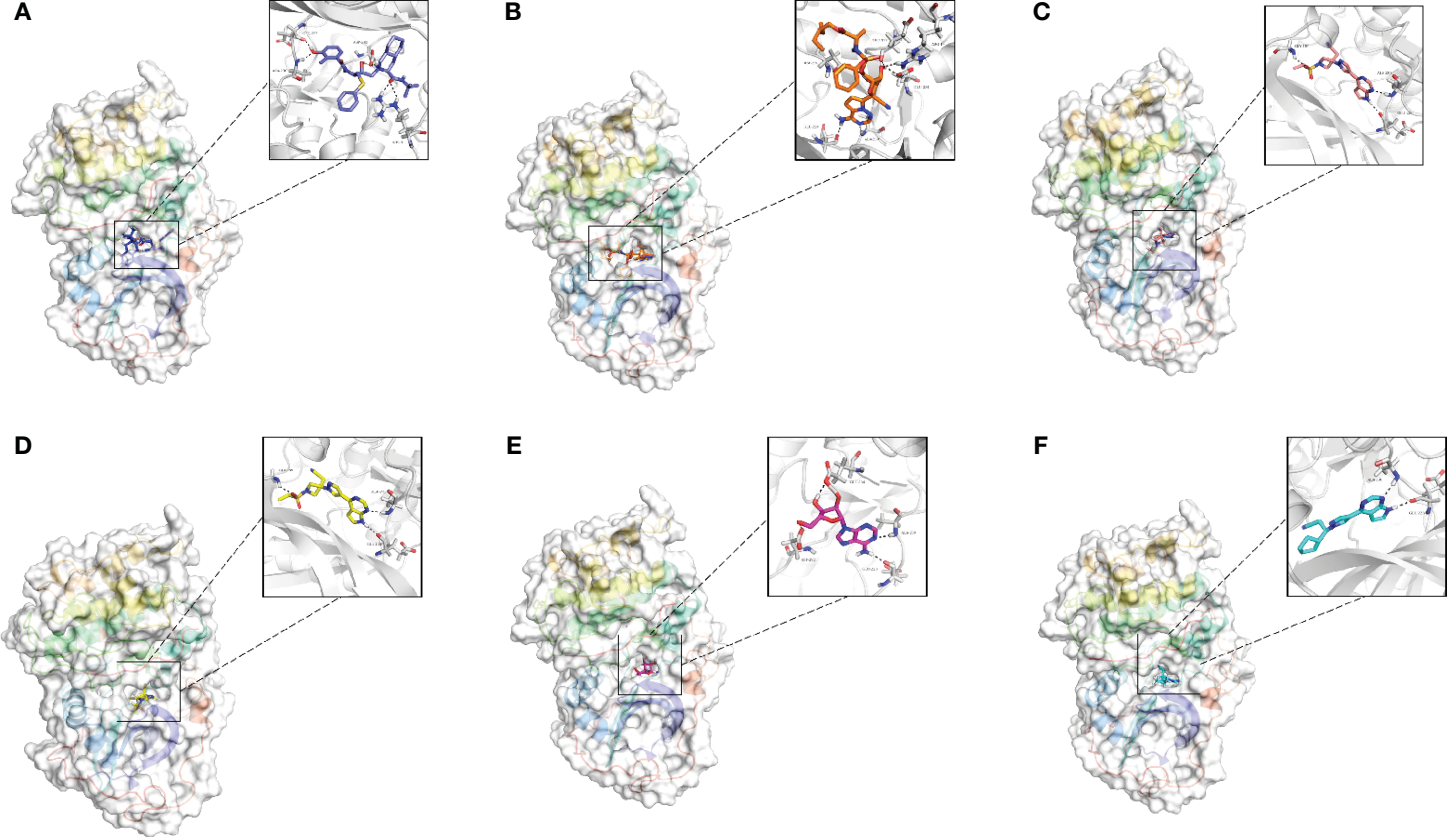
Molecular docking analysis of the top six ligands against the target protein AKT-1. **(A)** Surface interaction and molecular interaction of Nelfinavir in the active binding site of the AKT-1 protein. **(B)** Surface interaction and molecular interaction of Remdesivir in the active binding site of the AKT-1 protein. **(C)** Surface interaction and molecular interaction of Baricitinib in the active binding site of the AKT-1 protein. **(D)** Surface interaction and molecular interaction of Baricitinib phosphate in the active binding site of the AKT-1 protein. **(E)** Surface interaction and molecular interaction of Adenosine in the active binding site of the AKT-1 protein. **(F)** Surface interaction and molecular interaction of Ruxolitinib phosphate in the active binding site of the AKT-1 protein.

A total of 1729 FDA approved drugs were docked against the target protein AKT1 and ranked from the lowest to highest docking scores (**Table S5**). The top 6 compounds with the most favorable (lowest) docking scores were selected for further analysis (**Figures 7A–F**). These included Nelfinavir, Remdesivir, Baricitinib, Baricitinib phosphate, Adenosine, and Ruxolitinib phosphate (**Table 2**). To elucidate binding mechanisms, protein–ligand interaction analysis was conducted for the 6 top-ranking compounds using PyMOL visualization. The analysis revealed key interactions with Asp292, Glu234, Ala230, Glu228, Arg4, and other residues (**Figure 7**). Of note, the dietary supplement adenosine was among the top hits, representing a potentially preventive drug candidate with low toxicity.

**Table 2:** Docking scores of top 6 hit FDA-approved drugs.

Compound Name	Docking Score	Molecular formula	FDA Application	Disease
Nelfinavir	-10.733	C32H45N3O4S	20778	Immune system
Remdesivir	-9.309	C27H35N6O8P	214787	Immune system; Infection
Baricitinib	-9.183	C16H17N7O2S	207924	Inflammation
Baricitinib phosphate	-9.183	C16H20N7O6PS	207924	Inflammation
Adenosine	-9.017	C10H13N5O4	76404	Cardiovascular system
Ruxolitinib phosphate	-8.697	C17H21N6O4P	202192	Cardiovascular system

## 4 Discussion

In the current study, we utilized genetic variants from large-scale GWAS datasets in a two-sample MR analysis to demonstrate a causal relationship between obesity and gastric cancer risk. We further delineated potential molecular signatures linking elevated BMI to higher gastric cancer odds through integrated bioinformatics, meta-analyses of multi-omics data and multi-center cohort validation. Structure-based virtual screening was also performed to identify prospective FDA-approved drugs targeting the identified mediating mechanisms. Overall, our study provides unique insights into potentially modifiable drivers of the obesity-gastric cancer connection, while informing preventive strategies and novel therapeutics.

Adipose tissue inflammation may significantly contribute to cancer development and progression (30). In obesity, macrophages accumulation in in adipose tissue initiates a cascade immune cells interactions, proinflammatory cytokines, and hypoxic signaling that creates conditions enabling tumorigenesis (31). As the adipose tissue outgrows its blood supply, hypoxia causes adipocyte stress and death (32). inflammation is increasingly recognized as a pivotal factor in tumor progression, with cancers often originating from sites of infection, chronic irritation, and inflammation (33). Chronic tissue damage, such as inflammation in adipose tissue, can stimulate wound healing mechanisms that generate an oncogenic microenvironment. Interestingly, malignant cells may hijack inflammatory tissue repair processes to drive growth and invasion (30). A growing body of evidence indicates a close relationship between cancer development and inflammatory responses (34). Previous studies have also confirmed that both IL-6 and TNF genes are closely associated with immune inflammation (35, 36).

Inflammatory adipocytes release proinflammatory including cytokines such as TNFα, monocyte chemoattractant protein-1 (MCP-1), IL-1β, and IL-6 (37). Prior studies have indicated that TNFα, IL-1β, IL-6 promote tumor growth in obese mouse models. These cytokines can trigger inflammation and activate the oncogenic transcription factor STAT3 (38). Moreover, inflammation is associated with elevated circulating levels of C-reactive protein (CRP) and IL-6 (39). A previous study has reported that inflammation in adipose tissue is a crucial element in the development of obesity-induced insulin resistance and obesity-related metabolic diseases (40). Obesity-associated WAT inflammation was shown to correlate with mechanical modifications in the extracellular matrix (ECM), which can promote tumor growth (30). Insulin can stimulate the synthesis of IGF-I, both of which have strong mitogenic effects on tumor cells. For example, insulin and IGF-I activate PI3K/Akt/mTOR and Ras/Raf/MAPK signaling pathways, thereby stimulating tumor growth (41). It is becoming increasingly evident that the tumor microenvironment is majorly orchestrated by inflammatory cells and plays a pivotal role in the tumor process, including cell proliferation, survival, and migration (42). Adipose inflammation is reversible, representing a therapeutic target to potentially sever obesity-cancer links. Consequently, inhibiting inflammatory and proliferative pathways warrants exploration for combating obesity-driven carcinogenesis.

Obesity may also influence the development of cancer through dysfunctional adipose tissue and dysregulated signaling pathways that lead to altered mRNA expression profiles. Predominantly, these signaling pathways include PI3K/Akt, Ras/MAPK, and STAT3 signaling pathways, which are impacted by the cancer risk factors associated with obesity (43). Adipose tissue functions as an endocrine organ that produces and secretes polypeptide hormones and adipokines. Leptin and adiponectin are most abundant among these and are implicated in cancer development. Leptin, in particular, has been extensively investigated as a potential mediator of obesity-related cancers (44, 45). It is known to accelerate cancer progression by activating the PI3K, MAPK, and STAT3 signaling pathways (46). Therefore, targeting these pathways may provide a new approach to mitigating obesity-related cancer risks. The PI3K/Akt/mTOR signaling pathway is a key pathway linking obesity and cancer. It is the target of obesity and regulates cell proliferation and survival (26), thereby promoting tumor growth and metastasis (47). Notably, the PI3K/Akt/mTOR signaling pathway is also one of the signaling mediators of obesity-related factors and has thus become the focus of obesity and cancer. This pathway gets activated by insulin (48) and IGF-I, which are frequently found at high levels in the serum of overweight and obese patients, leading to enhanced PI3K/Akt/mTOR activation (49). The STAT3 pathway is widely studied and plays a crucial role in IL-6-mediated carcinogenesis, which can be reduced by blocking the IL-6 pathway (50). However, it is also activated by other signaling pathways that induce elevated oncogenic levels, making the relationship between obesity and cancer more complex.

Our study identified the dietary supplement adenosine as a potentially preventive, low toxicity drug candidate for mitigating the obesity-to-cancer transition. Adenosine exerts diverse biological effects via multiple receptors. These receptors are expressed on most immune cells and suppress immune/inflammatory responses, providing a protective shield to cells and tissues against an excessive immune reaction and immune-related damage (51). A few reports indicate adenosine receptors play roles in glucose homeostasis, inflammation, lipid synthesis, insulin resistance, and thermogenesis, suggesting that adenosine involvement in obesity pathogenesis (52, 53). Therefore, pharmacological modulation of adenosine receptors may have therapeutic potential.

## 5 Conclusion

In this study, we demonstrate a significant causal relationship between elevated body fat percentage and increased risk of gastric cancer using Mendelian randomization analysis. Further integrated bioinformatics and meta-analysis of multi-omics data and clinical evidence points to interconnected inflammatory and immune dysfunction centered around AKT1, IL-6, and TNF as key mechanisms linking obesity to elevated gastric cancer risk. Structure-based screening identified the dietary supplement adenosine as a promising safe AKT1 inhibitor, potentially mitigating the obesity-to-cancer transition. These findings could inform strategies to curb rising obesity-associated gastric cancer rates worldwide. Further experimental and clinical evaluation of adenosine for gastric cancer prevention in obese individuals is warranted.

## Data availability statement

The datasets presented in this study can be found in online repositories. The names of the repository/repositories and accession number(s) can be found in the article/**Supplementary Material**.

## Ethics statement

The studies involving humans were approved by Shengjing Hospital of China Medical University Affiliated Hospital of Liaoning University of Traditional Chinese Medicine. The studies were conducted in accordance with the local legislation and institutional requirements. The participants provided their written informed consent to participate in this study.

## Author contributions

AX and HT have contributed equally to this work and share first authorship. AX, HT, and KL conceived and designed this work. AX, SL, XZ and HT integrated and analyzed the data. AX, XZ, LY, HT, and KL plotted the figures. AX and HT wrote this manuscript. LY and KL revised the manuscript and provided suggestions for data analysis. Interpretation of the findings was done by AX, HT, SL, and KL. All authors contributed to the article and approved the submitted version. All the authors contributed substantially to the work.

## Glossary

**Table:**

sAKT1	AKT serine/threonine kinase 1
BH	Benjamini Hochberg
BLCA	Bladder Cancer
BMI	Body Mass Index
BP	Biological Process
BioGRID	Biological General Repository for Interaction Datasets
CC	Cellular Component
CESC	Cervical Cancer
CHOL	Bile Duct Cancer
COAD	Colon Cancer
COADREAD	Colon and Rectal Cancer
CRP	C-reactive protein
CTD	Comparative Toxicogenomics Database
DEG	differentially expressed gene
DLBC	Large B-cell Lymphoma
ESCA	Esophageal Cancer
GC	Gastric Cancer
GEO	Gene Expression Omnibus
GErel	gene expression interaction
GO	Gene Ontology
GPL	GEO Platform
H. pylori	Helicobacter pylori
HBV	Hepatitis B virus
HPRD	Human Protein Reference Database
IGF	insulin like growth factor
IGF-IR	type 1 insulin-like growth factor receptor
IL	interleukins
IL6	Interleukin 6
KEGG	Kyoto Encyclopedia of Genes and Genomes
KIRP	Kidney Papillary Cell Carcinoma
LIHC	Liver Cancer
MCODE	Molecular Complex Detection
MCP-1	Monocyte Chemoattractant Protein-1
MF	Molecular Function
NHOGC	Network of Human Obesity and Gastric Cancer
NK cells	natural killer cells
NS	not significant
OB	Obesity
OMIM	Online Mendelian Inheritance in Man
PCOS	Polycystic Ovarian Syndrome
PPrel	protein-protein interaction
PTM	post-translational modification
READ	Rectal Cancer
SKCM	Melanoma
STAD	Stomach Cancer
THYM	Thymoma
TNF	Tumor Necrosis Factor
TTD	Therapeutic Target Database
UCEC	Endometrioid Cancer
UCS	Uterine Carcinosarcoma
UVM	Ocular melanomas
WAT	white adipose tissue
WHO	World Health Organization
WBC	White Blood Cell Count
%NEUT	Percentage of Neutrophils
%EOS	Percentage of Eosinophils
%BASO	Percentage of Basophils
%MONO	Percentage of Monocytes
%LYMPH	Percentage of Lymphocytes
#NEUT	Neutrophil Count
#EOS	Eosinophil Count
#BASO	Basophil Count
#MONO	Monocyte Count
#LYMPH	Lymphocyte Count
RBC	Red Blood Cell Count
HGB	Hemoglobin
HCT	Hematocrit
MCV	Mean Corpuscular Volume
MCH	Mean Corpuscular Hemoglobin
MCHC	Mean Corpuscular Hemoglobin Concentration
RDW-CV	Red Cell Distribution Width Coefficient of Variation
RDW-SD	Red Cell Distribution Width Standard Deviation
PLT	Platelet Count
PCT	Platelet Crit
MPV	Mean Platelet Volume
PDW	Platelet Distribution Width
P-LCR	Platelet Large Cell Ratio
ECM	extracellular matrix
IV	Instrumental Variable
SNP	Single Nucleotide Polymorphism
IVW	Inverse Variance Weighted
OR	Odds Ratio
CI	Confidence Interval
GWAS	Genome-wide association studies
MR	Mendelian Randomization
LD	Linkage Disequilibrium
SBVS	structure-based virtual screening
