# Supplementary material for: The causal association between obesity and gastric cancer and shared molecular signatures: a large-scale Mendelian randomization and multi-omics analysis
Source: Front Oncol. 2023 Oct 26;13:1091958. doi: 10.3389/fonc.2023.1091958 (PMC10639150; doi:10.3389/fonc.2023.1091958)
Supplement: Supplementary file 1 [file DataSheet_1.docx]

Supplementary Material

1. **Supplementary Method**

**GEO database**

The GSE9624 dataset was submitted by Aguilera CM. This dataset contains the expression profile data of omental adipose tissue of 5 obese patients and 6 normal controls. The microarray platform is GPL570, namely Affymetrix Human Genome U133 Plus 2.0 Array; The GSE15653 dataset was submitted by Pihlajamäki J, from which we selected expression profile data of liver tissue contain 4 non-type II diabetic obese patients and 5 normal controls. The microarray platform is GPL96, namely Affymetrix Human Genome U133A Array. The GSE54129 dataset was submitted by Liu B, containing expression profile data of 111 human gastric cancer tissue and 21 non-cancerous gastric tissue, on GPL570 microarray platform; The GSE118916 dataset was submitted by Li L, containing the expression profile data of 15 human gastric cancer tissue and 15 non-cancerous gastric tissue. The microarray platform is GPL15207, namely Affymetrix Human Gene Expression Array; The GSE29998 dataset was submitted by Holbrook JD, containing the expression profile data of 50 human gastric cancer tissue and 49 non-cancerous gastric tissue. The microarray platform is GPL6947, namely Illumina HumanHT-12 V3.0 expression beadchip; The GSE79973 dataset, submitted by Shao Q, containing the expression profile data of 10 human gastric adenocarcinoma tissue and 10 normal tissue, also with GPL570 as the microarray platform.

**TCGA database**

To further compare the expression differences of these three genes in obese and normal population, we downloaded the gene expression profile (pancan normalized) data of all types of cancer and selected the expression profile data of the normal only. By downloading clinical phenotype data for each type of cancer, we found that only 15 types of cancer contained height and weight information. These cancers include Bladder Cancer (BLCA), Cervical Cancer (CESC), Bile Duct Cancer (CHOL), Colon Cancer (COAD), Colon and Rectal Cancer (COADREAD), Large B-cell Lymphoma (DLBC), Esophageal Cancer (ESCA), Kidney Papillary Cell Carcinoma (KIRP), Liver Cancer (LIHC), Rectal Cancer (READ), Melanoma (SKCM), Thymoma (THYM), Endometrioid Cancer (UCEC), Uterine Carcinosarcoma (UCS) and Ocular melanomas (UVM).

1. **Supplementary Figures**


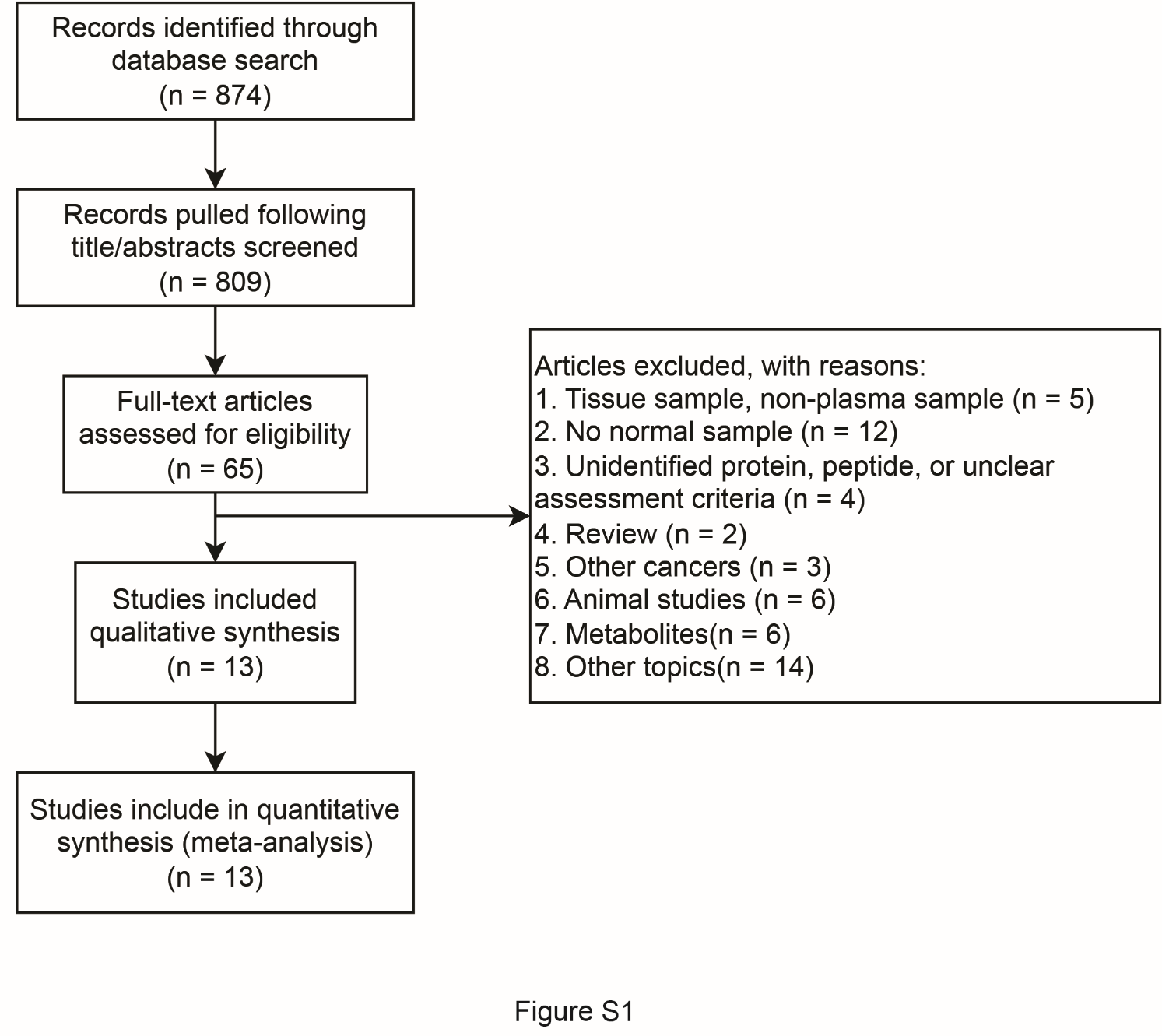


FIGURE S1 Flow chart of the selection process of the studies included in the meta-analysis of published plasma/serum proteomics data for obesity. A total of 13 studies containing 1662 individuals (880 with obesity and 782 controls) were included in our analysis.


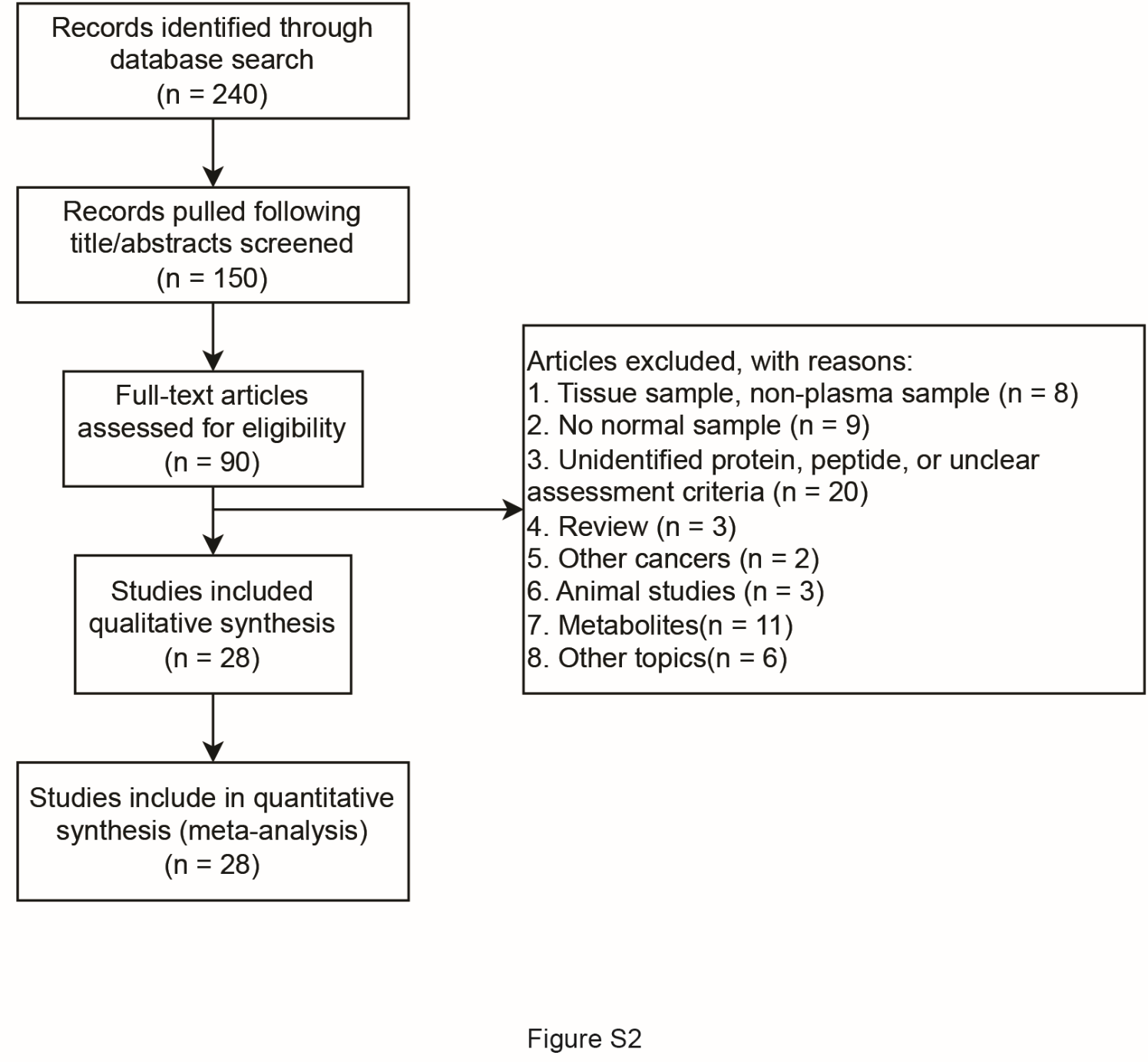


FIGURE S2 Flow chart of the selection process of the studies included in the meta-analysis of published plasma/serum proteomics data for gastric cancer. A total of 28 studies containing 3153 individuals (1678 with gastric cancer and 1475 controls) were included in our analysis.


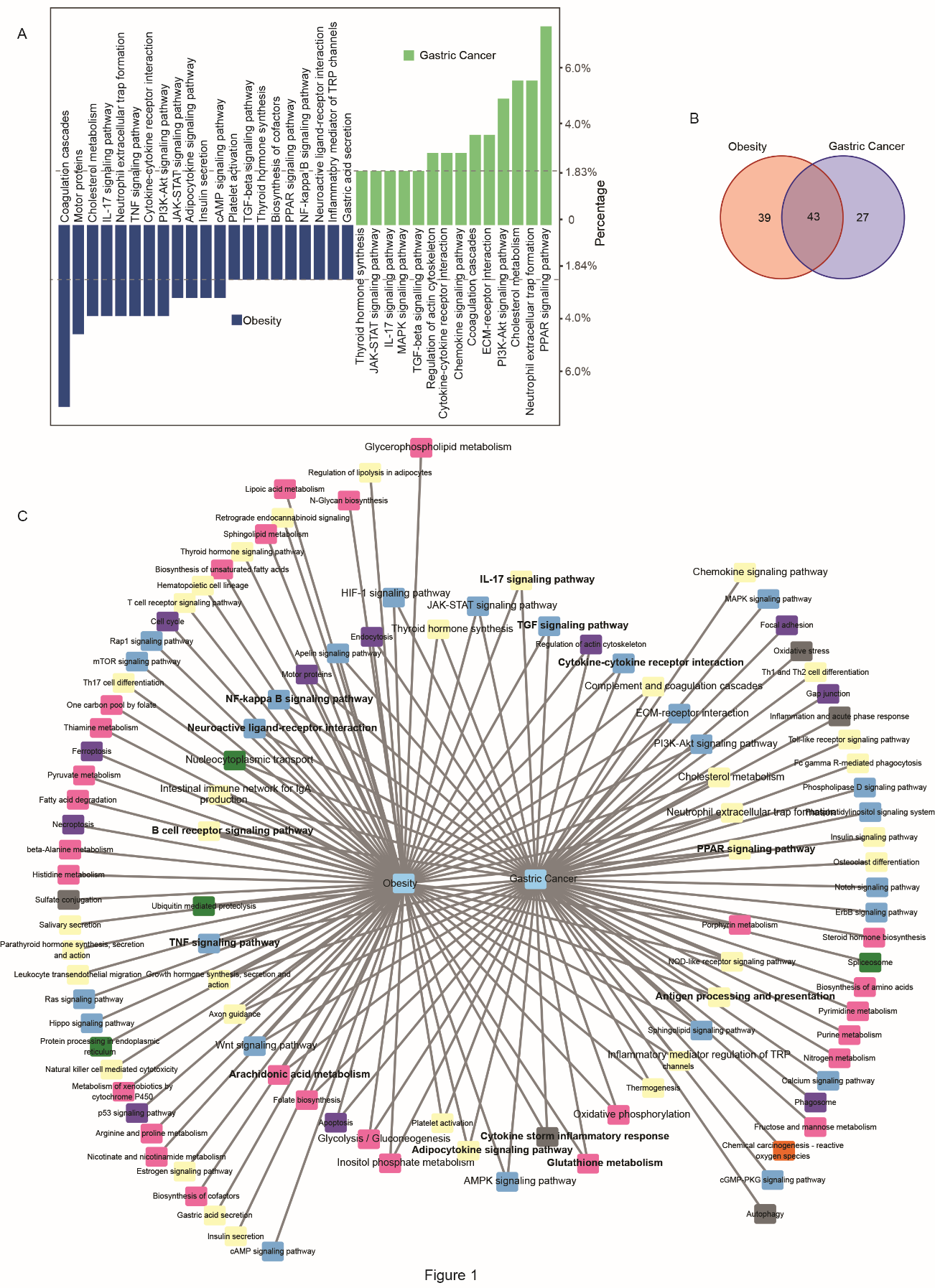


FIGURE S3 Meta-analysis of plasma/serum proteomics data identifies shared protein abnormalities between obesity and gastric cancer. (A) The significantly altered protein pathways in patients with obesity or gastric cancer compared to the controls. Enrichment analysis was performed with all the significantly altered proteins in the serum/plasma of the patients. The enrichment percentage = the number of significantly altered proteins in the enriched pathway/all the significantly changed proteins. (B) The number of shared proteins that were significantly altered in patients with obesity and gastric cancer. (C) The shared protein pathways that were significantly altered in both obese patients and patients with gastric cancer (The inner cycle in the interactome network). Note: A total of 13 studies containing 1662 individuals were included in the meta-analysis of proteomic changes in obesity, and a total of 28 studies containing 3153 individuals were included in the analysis of proteomic changes in gastric cancer.


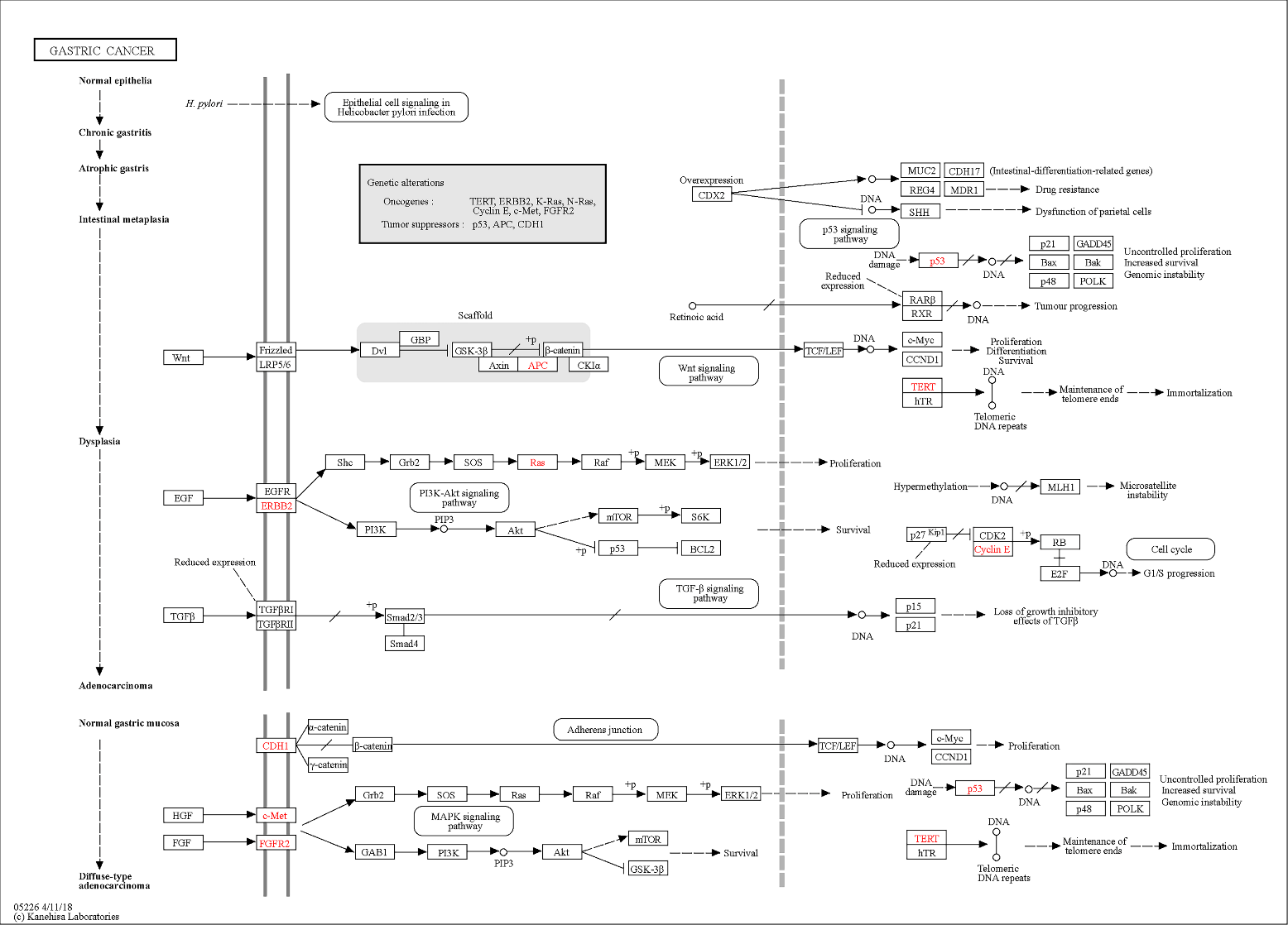


FIGURE S4 The shared KEGG pathways between obesity and gastric cancer.


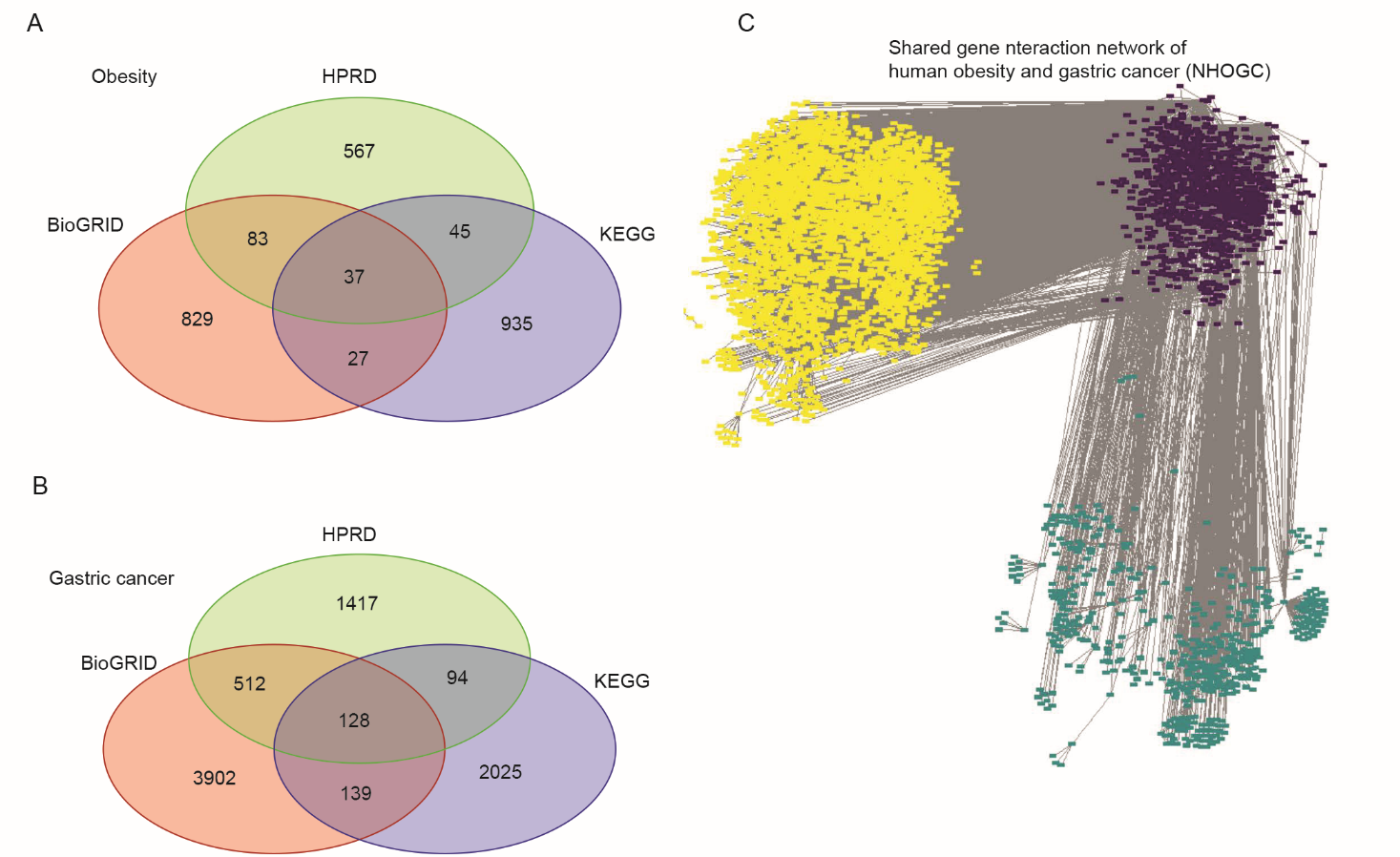


FIGURE S5 Distribution of gene interactions in the Network of Human Obesity and Gastric Cancer (NHOGC).

(A) Venn diagram of the source of interaction information in the obesity network (Number of edges in the network). (B) Venn diagram of the source of interaction information in gastric cancer (Number of edges in the network). (C) The construction of an integrated gene interaction network of human obesity and gastric cancer (NHOGC).

Notes: For Figure S4C, green indicates obesity-associated genes, yellow indicates gastric cancer-associated genes, and Purple indicates the disease-associated genes shared by both.


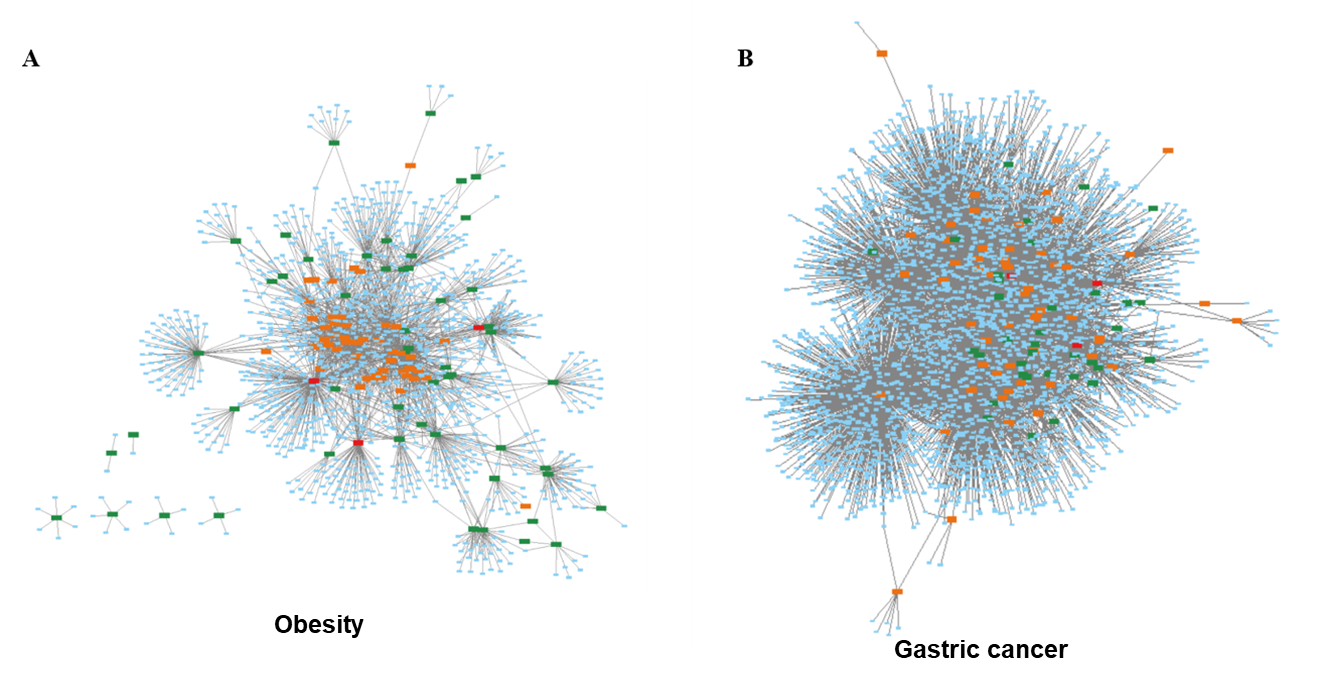


FIGURE S6 The subnetworks of bioactive modules in the integrated gene interaction network of obesity and gastric cancer (NHOGC). (A) Obesity; (B) Gastric cancer.

Green indicates obesity-associated genes; Orange indicates gastric-cancer-associated genes; Red indicates the disease-associated genes shared by both.

1. **Supplementary Tables (Single Excel files)**

**Table S1** The characteristics of included studies for meta-analysis of plasma serum/plasma proteome in obese patients.

**Table S2** The characteristics of included studies for meta-analysis of plasma serum/plasma proteome in patients with gastric cancer

**Table S3** Strongly correlated instrumental variables in body fat percentage (*P* < 5×10^−8^).

**Table S4** Strongly correlated instrumental variables in body fat percentage with F-statistic.

**Table S5** Compounds with their docking scores.
